# Applications of Fog Computing in Healthcare

**DOI:** 10.7759/cureus.64263

**Published:** 2024-07-10

**Authors:** Naveen Jeyaraman, Madhan Jeyaraman, Sankalp Yadav, Swaminathan Ramasubramanian, Sangeetha Balaji, Sathish Muthu, Chithra Lekha P, Bishnu P Patro

**Affiliations:** 1 Orthopaedics, ACS Medical College and Hospital, Dr. MGR Educational and Research Institute, Chennai, IND; 2 Clinical Research, Virginia Tech India, Dr. MGR Educational and Research Institute, Chennai, IND; 3 Medicine, Shri Madan Lal Khurana Chest Clinic, New Delhi, IND; 4 Orthopaedics, Government Medical College, Omandurar Government Estate, Chennai, IND; 5 Orthopaedics and Traumatology, Orthopaedic Research Group, Coimbatore, IND; 6 Biotechnology, Karpagam Academy of Higher Education, Coimbatore, IND; 7 Orthopaedics, Government Medical College, Karur, IND; 8 Orthopaedics, All India Institute of Medical Sciences, Bhubaneswar, IND

**Keywords:** artificial intelligence, data security, medical diagnostics, real-time data processing, healthcare technology, fog computing

## Abstract

Fog computing is a decentralized computing infrastructure that processes data at or near its source, reducing latency and bandwidth usage. This technology is gaining traction in healthcare due to its potential to enhance real-time data processing and decision-making capabilities in critical medical scenarios.

A systematic review of existing literature on fog computing in healthcare was conducted. The review included searches in major databases such as PubMed, IEEE Xplore, Scopus, and Google Scholar. The search terms used were "fog computing in healthcare," "real-time diagnostics and fog computing," "continuous patient monitoring fog computing," "predictive analytics fog computing," "interoperability in fog computing healthcare," "scalability issues fog computing healthcare," and "security challenges fog computing healthcare." Articles published between 2010 and 2023 were considered. Inclusion criteria encompassed peer-reviewed articles, conference papers, and review articles focusing on the applications of fog computing in healthcare. Exclusion criteria were articles not available in English, those not related to healthcare applications, and those lacking empirical data. Data extraction focused on the applications of fog computing in real-time diagnostics, continuous monitoring, predictive analytics, and the identified challenges of interoperability, scalability, and security.

Fog computing significantly enhances diagnostic capabilities by facilitating real-time data analysis, crucial for urgent diagnostics such as stroke detection, by processing data closer to its source. It also improves monitoring during surgeries by enabling real-time processing of vital signs and physiological parameters, thereby enhancing patient safety. In chronic disease management, continuous data collection and analysis through wearable devices allow for proactive disease management and timely adjustments to treatment plans. Additionally, fog computing supports telemedicine by enabling real-time communication between remote specialists and patients, thereby improving access to specialist care in underserved regions.

Fog computing offers transformative potential in healthcare, improving diagnostic precision, patient monitoring, and personalized treatment. Addressing the challenges of interoperability, scalability, and security will be crucial for fully realizing the benefits of fog computing in healthcare, leading to a more connected and efficient healthcare environment.

## Introduction and background

Digital technologies have significantly reshaped healthcare delivery, introducing innovative methods that enhance diagnostics and patient care. Among these advancements, fog computing has emerged as a key technological paradigm, pushing data processing closer to where data originates, namely, at the network's edge [1,2]. This approach contrasts sharply with traditional cloud computing (Table 1), which relies on centralized data centers.

**Table 1:** Comparison of cloud computing and fog computing in healthcare.

Feature	Cloud computing	Fog computing
Data processing location	Centralized in remote data centers	Decentralized, at or near the source of data
Latency	Higher due to longer distance data travels	Lower, as data are processed closer to their origin
Bandwidth usage	Higher, as large amounts of data are transmitted to and from the cloud	Lower, due to local data processing and reduced data transmission
Real-time capability	Limited by network latency	Enhanced, suitable for time-sensitive applications such as emergency response and critical care monitoring
Security risks	Higher, due to extensive data transmission and centralized storage	Reduced, with data processed and often stored locally, minimizing exposure to cyber threats
Interoperability	Dependent on internet and cloud services' protocols	Requires local interoperability standards but is less dependent on external networks
Scalability	Easily scalable with cloud resources	Scalability depends on local infrastructure capabilities
Cost	Potentially lower upfront, with ongoing operational expenses	Higher upfront costs for local infrastructure, potentially lower operational costs

**Figure 1:**
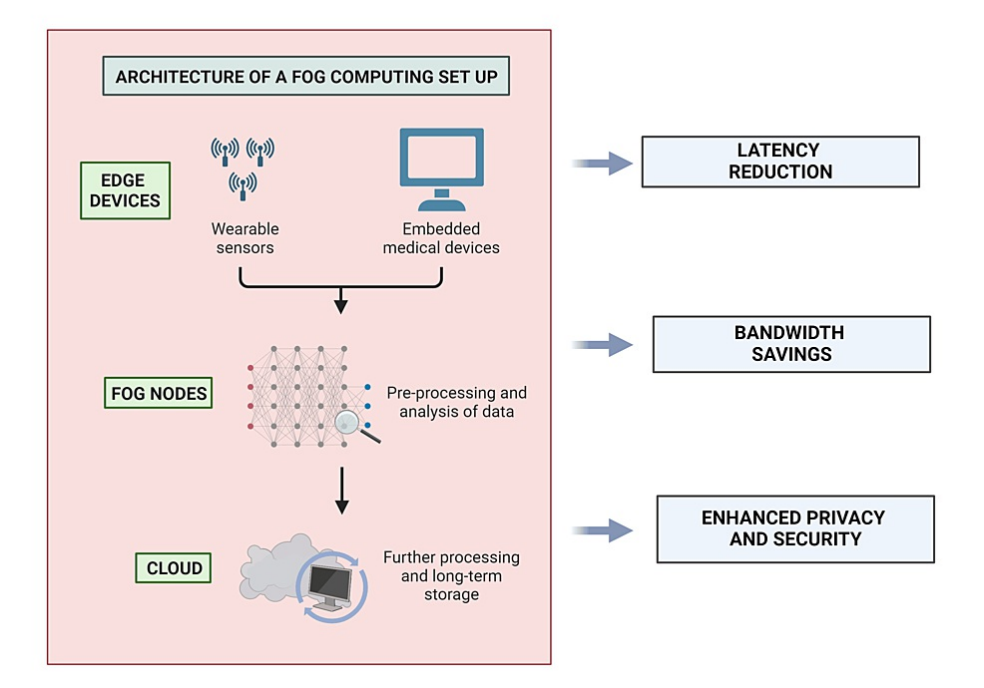
Architecture and advantages of fog computing model.

By minimizing the distance data must travel for processing, fog computing effectively reduces latency and bandwidth use, crucial for real-time healthcare applications [3]. These characteristics are particularly valuable in critical medical scenarios such as emergency response, continuous monitoring in intensive care units, and during complex surgical procedures where immediate data analysis is paramount [4-6]. The advantages and the architecture of the fog computing model are given in Figure 1 [7].

Fog computing, an extension of cloud computing, decentralizes data processing and storage by bringing computational resources closer to the data source. In healthcare, fog computing enables real-time data processing, enhancing patient care through swift decision-making and continuous monitoring. By positioning computational resources at the network edge, it reduces latency, which is crucial for time-sensitive medical applications such as remote patient monitoring, telemedicine, and emergency response systems. This architecture supports efficient data management and analysis from wearable devices, medical sensors, and hospital information systems. Additionally, fog computing enhances data security and privacy by minimizing data transmission to central servers, thereby reducing vulnerability to cyber threats. Its scalable nature accommodates the growing volume of healthcare data, ensuring robust, real-time analytics, and improved patient outcomes. Fog computing's integration in healthcare signifies a pivotal advancement toward a more responsive, secure, and efficient medical data ecosystem.

Despite the promising advantages of fog computing, its practical implementation in healthcare settings is hindered by several knowledge gaps. Firstly, there is an apparent lack of standardization across the diverse array of medical devices and data formats, which complicates the integration of fog computing solutions. Each device or system often operates independently without compatibility consideration, leading to potential issues in data coherence and system interoperability [8,9]. Secondly, scalability poses another significant challenge. The healthcare sector's data volume is expanding rapidly, driven by increases in the number of connected devices and the granularity of data collected. Fog computing systems must be capable of scaling to accommodate this growth without compromising data processing speed or system reliability [10,11]. Thirdly, security concerns are particularly acute in the healthcare sector due to the sensitive nature of personal health information. The decentralized processing inherent in fog computing creates multiple points of potential vulnerability, necessitating robust security protocols to safeguard against unauthorized access and data breaches [12,13].

The primary aim of this review is to systematically investigate and address the interoperability, scalability, and security challenges of fog computing in healthcare, with the objective of enhancing real-time data processing capabilities and improving patient care outcomes.

## Review

Enhancing diagnostic capabilities

Fog computing significantly enhances diagnostic capabilities in healthcare by enabling real-time data analysis and processing. This advanced technological framework is particularly crucial for urgent medical diagnostics, where rapid and accurate data analysis can be the difference between life and death [14]. By processing data closer to its source, fog computing reduces latency, improves response times, and enhances the accuracy of medical diagnostics, which is essential in critical care situations such as stroke detection and during surgical procedures [15,16].

Real-Time Data Processing

Stroke detection exemplifies an area where fog computing’s impact on diagnostics is particularly profound. Immediate diagnosis and intervention are critical for stroke victims, as the treatment window for effective intervention is exceedingly narrow. Typically, the first few hours after a stroke are crucial; delays in treatment can lead to significant and irreversible damage. Fog computing facilitates the real-time processing of data from CT scans, MRIs, and other monitoring devices directly at the site of patient care, whether in an ambulance or at the emergency room [17]. This enables healthcare providers to make quicker decisions about treatment strategies based on the rapid assessment of imaging and physiological data [18,19]. For example, in the case of ischemic stroke, where timely restoration of blood flow is necessary, fog computing can support real-time imaging analytics to assess the extent of a clot and guide thrombolytic procedures. This capability not only speeds up the diagnostic process but also enhances the precision with which treatments are administered, thereby improving patient outcomes.

Enhancing Monitoring Capabilities During Surgeries

During surgical procedures, the ability to monitor vital signs and other physiological parameters in real time is crucial for patient safety. Fog computing plays a critical role here by providing the computational power to process data from multiple monitoring devices simultaneously. This includes real-time analysis of electrocardiograms, blood pressure, oxygen saturation, and other critical metrics that must be closely watched during surgery. By leveraging fog computing, data from these devices can be processed locally, without the delays that cloud computing might introduce. This means anomalies are detected almost instantaneously, allowing for immediate response by the surgical team [20-24]. For instance, if a patient’s blood pressure drops suddenly during surgery, fog computing can help in quickly analyzing the trend data from the monitoring devices to alert the anesthesiologist, who can then take swift action to stabilize the patient [22].

Supporting Advanced Imaging Techniques

**Figure 2:**
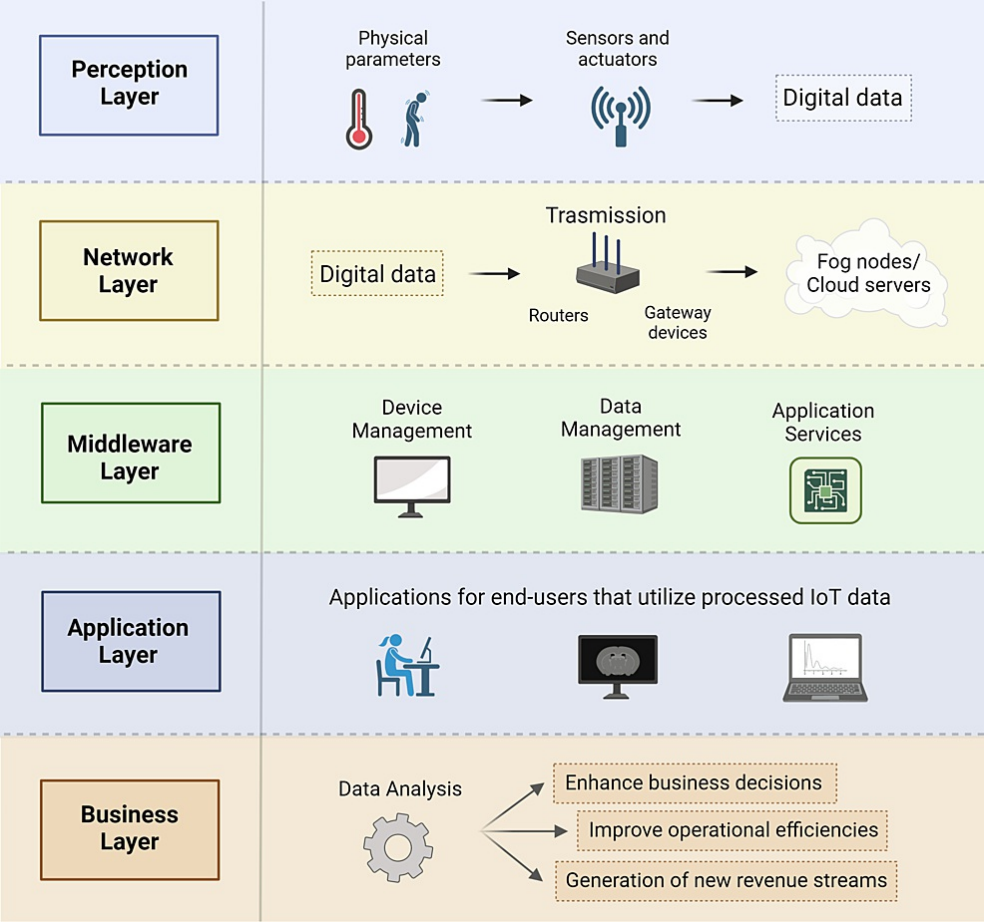
Layers of data transmission in fog computing model.

Advanced imaging techniques, such as intraoperative MRI, are increasingly used during complex surgeries, including those for cancer or neurological disorders. These imaging techniques generate large volumes of data that require rapid processing to be useful during a procedure. Fog computing provides the necessary infrastructure to process these large datasets locally, facilitating real-time imaging that guides surgical decisions [25-27]. This is particularly important in neurosurgery where slight deviations can have significant consequences. By processing imaging data on-site, surgeons can view updated scans quickly, allowing for precise modifications to their surgical approach based on the most current anatomical information. The layers involved in the data processing from input to the output stream in fog computing are given in Figure 2.

Facilitating Predictive Analytics

Fog computing also enhances diagnostic capabilities through predictive analytics, which can anticipate patient complications before they become critical. By analyzing historical data and real-time inputs from patient monitoring systems, fog computing enables predictive models that can forecast potential declines in patient health [16,28,29]. For instance, in critical care units, predictive analytics can help in anticipating septic shock or other complications, enabling preemptive treatment that can save lives. This application not only improves patient outcomes but also optimizes the use of healthcare resources.

Integrating With Telemedicine

Moreover, fog computing's capability extends to supporting telemedicine, especially in remote or underserved regions where immediate access to specialist care is not available. In scenarios such as telesurgery or remote diagnostics, fog computing can process diagnostic data locally, thereby facilitating real-time communication between local practitioners and remote specialists. This integration ensures that patients in remote locations receive expert care with the same speed and efficiency as those in advanced medical facilities [30-32].

Improving patient care

Continuous Monitoring in Chronic Disease Management

Chronic diseases, such as diabetes, heart disease, and asthma, require ongoing management and monitoring. Traditional approaches often rely on periodic visits to healthcare facilities, which can miss fluctuations in the patient's condition between appointments. Fog computing changes this dynamic by enabling continuous data collection through wearable devices and home monitoring equipment. These devices can measure vital signs, glucose levels, heart rhythms, and other relevant health metrics in real time. The data collected are processed locally, allowing for immediate responses and adjustments in patient care plans. For example, in the management of diabetes, continuous glucose monitors (CGMs) can transmit data to a fog computing system, which analyzes trends and can trigger alerts if glucose levels move outside of prescribed ranges. This system not only informs the patient and their healthcare provider but can also automatically adjust insulin doses delivered by a connected insulin pump, thus maintaining optimal glucose levels more effectively [33-35].

Enhancing Personalized Medicine

Personalized medicine, which tailors medical treatment to the individual characteristics of each patient, benefits significantly from fog computing [36-38]. This approach often requires the integration and analysis of large datasets, including genomic data, medical history, environmental factors, and lifestyle information. Fog computing facilitates the local processing of these diverse data types, enabling healthcare systems to create customized treatment plans that are dynamically adjusted based on real-time data inputs. In oncology, for example, personalized treatment plans based on genetic mutations can be adjusted in real time as new data from ongoing patient monitoring and lab results become available. Fog computing systems can quickly process these data, allowing oncologists to modify chemotherapy regimens or other treatments to optimize efficacy and minimize side effects, based on the patient’s current health status and treatment response [39-41].

Supporting Remote Patient Monitoring

Remote patient monitoring, crucial for patients living in rural or underserved areas, is another area where fog computing has a significant impact. By processing data locally, fog computing reduces the dependence on continuous internet connectivity, which can be unreliable in such regions. Patients with chronic conditions can use wearable devices that monitor their health metrics and send data to a nearby fog node, which processes the data and can alert healthcare providers if intervention is needed. This not only ensures timely medical attention but also increases the patient's ability to manage their health proactively [42-44]. For instance, heart failure patients can be monitored continuously with wearable devices that track heart rate, activity levels, and other vital signs. The fog computing system can analyze these data to predict potential exacerbations, enabling preemptive adjustments to treatment before a critical event occurs.

Facilitating Advanced Home Care

As healthcare shifts toward more home-based treatments, fog computing supports the advanced home care necessary for complex patient needs. Home healthcare devices, integrated through fog computing, can perform functions such as monitoring sleep patterns, medication adherence, and rehabilitation progress. These devices collect data that a fog node can analyze to provide insights into the patient’s recovery and health status [45]. Adjustments to medications or therapies can be made almost instantaneously based on these data, promoting better health outcomes and reducing the need for hospital readmissions.

Integrating Wearable Technologies

The integration of wearable technologies with fog computing significantly enhances patient engagement and self-management. Wearables that monitor physical activity, heart rate, and other health indicators provide patients with immediate feedback about their health status. This real-time feedback, processed through fog computing, empowers patients to make informed decisions about their daily activities and management of their conditions. It also facilitates a more collaborative approach to healthcare, where patients and providers can work together more closely to manage health conditions effectively [46-48].

Data management and security

Localized Data Processing for Enhanced Security

The traditional model of transmitting data to centralized cloud servers poses significant security risks, including potential data breaches and unauthorized access during transmission. Fog computing mitigates these risks by processing data locally, at or near the point of collection. This means that sensitive data, such as patient health records and real-time monitoring data, do not traverse the internet or other networks extensively, thereby reducing the exposure to potential cyber threats. Localized processing not only secures data but also complies with strict healthcare regulations like the Health Insurance Portability and Accountability Act (HIPAA) in the US or the General Data Protection Regulation (GDPR) in Europe. These regulations mandate rigorous data security measures and patient privacy protections, which are more controllable and enforceable when data remains on local devices or nearby nodes rather than being sent to remote servers [49-51]. For example, a hospital using fog computing can ensure that all patient data generated through medical devices are processed and analyzed within its local network, significantly limiting access to this sensitive information and reducing the risk of data breaches.

Efficient Data Management With Fog Computing

**Figure 3:**
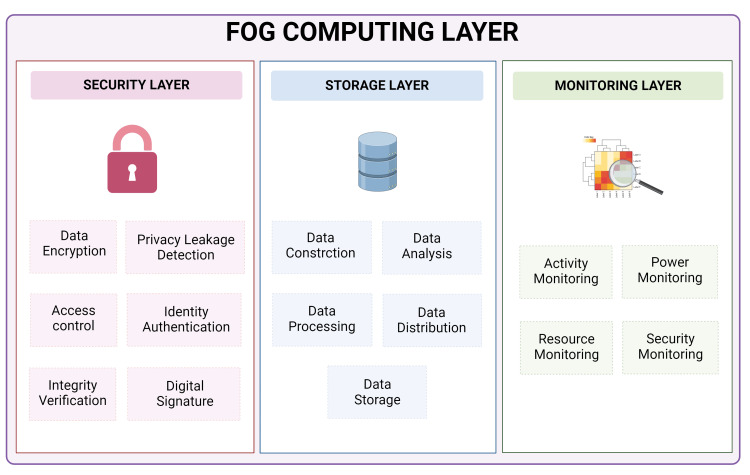
Events in each layer of the fog computing model.

Healthcare facilities generate vast amounts of data daily, from electronic health records and lab results to imaging data and real-time patient monitoring systems. Managing these data efficiently is crucial not only for patient care but also for operational management and medical research. Fog computing enhances data management by providing the computational power to handle large datasets at the network's edge [52,53]. This setup allows healthcare providers to quickly access and use these data for real-time decision-making and patient care without the latency involved in querying distant cloud servers. Moreover, fog computing supports scalable data infrastructure, which is adaptable to the increasing influx of data from various sources, including newer medical devices and Internet of Things (IoT) sensors. This scalability ensures that healthcare systems can continue to expand without the corresponding exponential increase in data management costs and complexity. For instance, a fog node can aggregate and analyze data from multiple sources and only send necessary or relevant information to the cloud for further analysis or long-term storage, thereby optimizing network and storage resources. Various actions performed in each layer of the fog computing model are given in Figure 3.

Real-Time Data Analytics for Immediate Insights

The capability of fog computing to support real-time data analytics further underscores its importance in healthcare. By analyzing data as they are generated, healthcare providers can gain immediate insights into patient conditions, potentially lifesaving in critical care scenarios [14,54,55]. For instance, real-time analysis of cardiac monitor data in a fog computing setup can immediately identify patterns indicative of an impending heart attack, allowing for rapid intervention. This immediate processing capability is crucial not only for emergency responses but also for routine care, where early detection of potential health issues can lead to more effective management and treatment strategies. It enhances the overall quality of care by enabling healthcare providers to act quickly based on the latest data, ensuring that patient care decisions are informed by the most current and comprehensive information available [14,56].

Enhancing Privacy Through Data Localization

In addition to security, privacy is a critical concern in healthcare. Patients expect their health information to be handled with the utmost confidentiality. Fog computing strengthens privacy by keeping data localized, and reducing the number of checkpoints through which data must pass, thus minimizing the visibility of sensitive information to unauthorized parties [57,58]. Local processing within a fog node means that detailed patient data can be anonymized or minimized before being transmitted to central servers for further analysis or storage, adhering to privacy best practices and regulations. Furthermore, fog computing enables healthcare organizations to implement robust access control mechanisms at the network edge. These controls can be finely tuned to limit access to sensitive data based on roles or requirements, ensuring that only authorized personnel can view or process patient information. This localized control is vital in environments where data sensitivity is high and the potential impact of unauthorized access could be severe [59-64]. The applications of fog computing in healthcare are tabulated in Table 2.

**Table 2:** Fog computing applications in healthcare and their impact.

Application	Description	Impact on healthcare
Real-time stroke detection	Processing data from imaging devices in real time, facilitating rapid diagnosis and treatment	Reduces time to treatment, critical for conditions like ischemic strokes, potentially reducing long-term disability
Surgical monitoring	Monitoring and analyzing vital signs and other parameters during surgeries in real time	Enhances patient safety by enabling immediate reactions to physiological changes
Continuous chronic disease management	Using wearable devices to monitor and manage chronic conditions like diabetes in real time	Improves disease management by providing constant feedback and allowing immediate adjustments in treatment
Telemedicine support	Local processing supports real-time communication between remote specialists and patients, facilitating teleconsultations	Expands access to specialist care, particularly in remote areas, improving outcomes by providing timely expert interventions
Advanced home care	Integration of fog computing with home health devices to monitor and manage post-hospitalization recovery	Reduces readmission rates by providing continuous, personalized monitoring and adjusting treatments based on real-time data

Challenges

Interoperability Challenges

**Figure 4:**
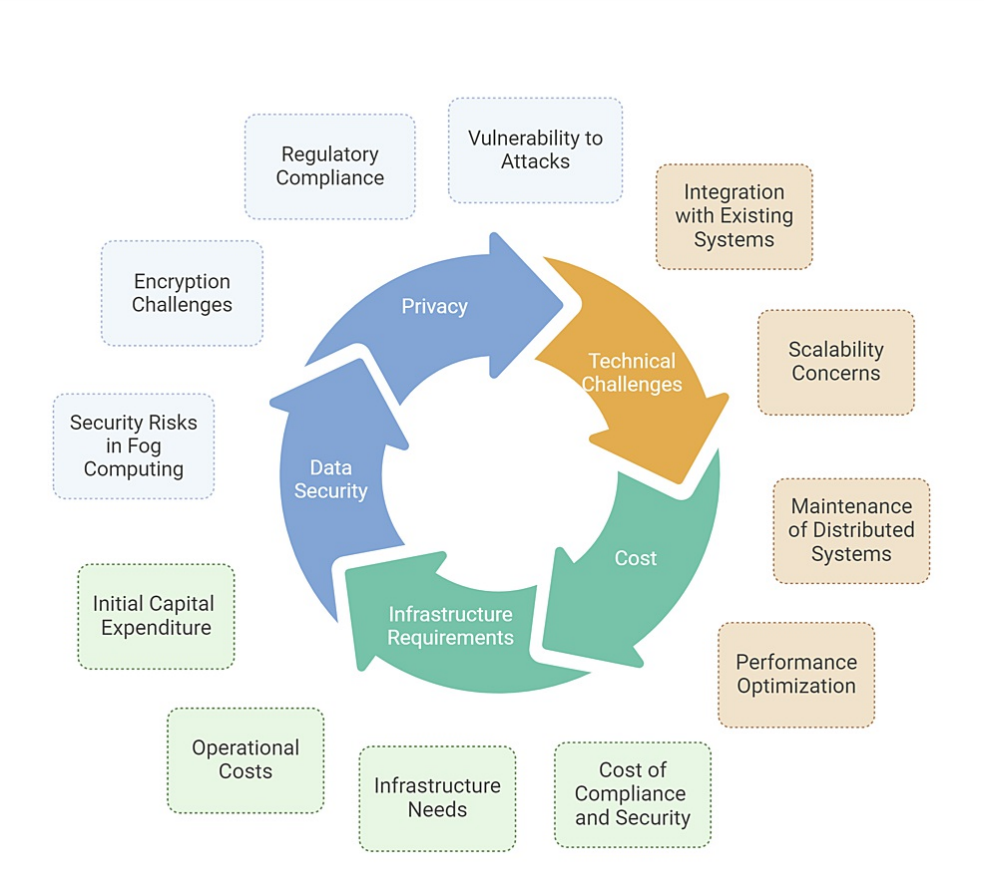
Challenges with fog computing in healthcare.

Healthcare systems typically employ a diverse array of devices, software systems, and data formats. Many of these systems are designed to function independently without considering integration with other technologies. This disparity makes it difficult to achieve seamless communication and data exchange between different devices and layers within the fog computing architecture. The lack of standardization across devices and protocols can lead to significant integration issues, which in turn can result in incomplete data analyses, errors, and increased costs [65]. For example, a wearable device from one manufacturer might use different communication protocols compared to another, or it might collect data in a format that is not readily compatible with the hospital's existing data systems as shown in Figure 4 and Table 3.

**Table 3:** Key challenges and proposed solutions for fog computing in healthcare. AI: artificial intelligence; ML: machine learning.

Challenge	Description	Proposed solutions
Interoperability	Difficulty in seamless integration of diverse medical devices and systems	Development and enforcement of universal standards for device communication and data formats
Scalability	Need to handle growing data volumes and device connectivity without performance degradation	Enhance local processing power and develop scalable network architectures
Security	High risk of data breaches and unauthorized access due to decentralized data processing	Implement advanced encryption, use blockchain technology for secure data transactions, and continuous security monitoring
Technical limitations	Limitations in local computational power, especially in remote or resource-limited healthcare settings	Deploy AI and ML to optimize data processing efficiency and manage computational loads
Maintenance	Managing and updating numerous decentralized nodes can be complex and resource-intensive	Utilize sophisticated management tools and skilled personnel, automate updates and maintenance routines

To address these challenges, there is a critical need for universal standards that ensure all devices and systems can communicate effectively, regardless of their make or model. This would require collaboration between technology developers, healthcare providers, and regulatory bodies to create and enforce these standards.

Scalability Issues

As the volume of data generated by healthcare devices continues to grow exponentially, the infrastructure supporting fog computing must also scale accordingly. This scaling involves not just physical infrastructure, such as servers and storage devices, but also the network architecture and software systems that manage and process data. Scaling fog computing solutions involves complex logistics and significant investment, especially in ensuring that data processing capabilities can handle large influxes of data without degradation in performance [65]. For instance, adding more devices to a system (such as in a large hospital) can strain the local fog nodes if they are not designed to scale efficiently. This can lead to slower data processing speeds and reduced reliability, which are unacceptable in clinical settings where decision-making speed and accuracy are critical.

Technical Limitations

These limitations often pertain to the computational power available at the edge of the network, security concerns, and the maintenance and management of the fog computing infrastructure. Local data processing requires substantial computational resources, which might not be feasible for smaller healthcare providers or in remote locations. The energy consumption associated with operating powerful local servers also poses environmental and economic concerns, particularly if the energy needs to be sustained 24/7 to support critical healthcare operations. While fog computing enhances data security by localizing data processing, it also introduces new security challenges. Each node in a fog computing architecture represents a potential entry point for cyber threats. Ensuring robust security across numerous decentralized nodes increases complexity and requires sophisticated security protocols and constant vigilance to guard against breaches. Maintaining and managing a distributed fog computing system involves significant operational challenges [65]. The administration of multiple fog nodes, each potentially running different applications and systems, can be cumbersome and resource-intensive. Ensuring consistent updates, managing system failures, and troubleshooting issues across a dispersed infrastructure require advanced management tools and skilled personnel, which can be a strain on healthcare providers' resources.

Future directions

The ongoing development of universal standards for interoperability marks a pivotal direction in the realm of fog computing within healthcare. As this sector transitions into a more cohesive ecosystem of interconnected devices and systems, establishing uniform protocols is crucial. Anticipated advancements may involve the formulation of open platforms where both device manufacturers and software providers conform to standardized communication and data norms. Such standardization would enable seamless data exchanges and integration across diverse devices and systems, thus elevating the utility and operational efficiency of fog computing in clinical contexts. These initiatives necessitate a collaborative approach among technology developers, healthcare providers, and regulatory authorities to balance clinical demands with innovation. The incorporation of artificial intelligence (AI) and machine learning (ML) within fog computing stands to transform healthcare service delivery profoundly. By deploying AI and ML at the network's edge, real-time data processing can yield instant insights into patient health, predicting potential issues before they escalate. For instance, systems enhanced with AI could monitor data from wearable devices continuously, identifying early symptoms of heart failure or other chronic ailments, which facilitates preventative care or timely medical interventions. Additionally, AI can refine fog computing network operations by dynamically managing resources and optimizing computational load distribution, thereby enhancing performance and reducing both operational costs and energy usage.

Enhanced security measures are essential, given the sensitive nature of personal health information. Future enhancements in fog computing should concentrate on advancing security protocols to defend against increasingly complex cyber threats. Techniques such as sophisticated encryption, blockchain technology, and perpetual security monitoring tailored for fog environments could be pivotal. Blockchain technology, in particular, shows considerable promise for securing transactions and data exchanges across decentralized networks, ensuring both data integrity and traceability. Integrating blockchain could facilitate secure, transparent management of patient records, allowing data sharing among authorized parties while blocking unauthorized access. Personalized healthcare services represent another significant potential advancement in fog computing, utilizing the extensive data generated by the Internet of Medical Things (IoMT). Future systems might employ advanced analytic tools to deliver personalized health recommendations directly to patients through their devices, based on real-time data analysis that encompasses medical, lifestyle, environmental, and genetic information. This comprehensive approach to health management could be complemented by personalized virtual health assistants, which would process data locally to offer real-time health advice, medication reminders, and direct interactions with healthcare providers, thereby improving patient engagement and adherence to treatment plans. Optimizing resource allocation within healthcare facilities is a likely focus for future advancements in fog computing. By analyzing data from various sources, such as patient admissions, treatment outcomes, and operational metrics, fog computing can assist healthcare administrators in optimizing staffing, managing equipment use, and minimizing wait times. This enhances both care quality and overall operational efficiency. Environments like smart hospitals, powered by fog computing, could automatically adjust resources in response to patient influx and hospital capacity, using predictive analytics to forecast future demands and ensuring resource use remains efficient while maintaining high care standards.

## Conclusions

Fog computing presents a transformative opportunity in healthcare, enhancing diagnostic precision, real-time patient monitoring, and personalized treatment. By processing data closer to its source, fog computing addresses critical latency and bandwidth challenges, making it ideal for time-sensitive applications such as emergency responses and complex surgeries. The decentralized architecture bolsters security by minimizing data exposure to cyber threats and enhances patient privacy through localized data processing. Nevertheless, significant challenges persist, including the necessity for interoperability standards, scalability solutions, and robust security protocols to address the intricacies of decentralized systems. As healthcare systems increasingly integrate fog computing, future developments must focus on universal interoperability standards, leveraging AI and ML for dynamic resource management and predictive analytics, and advancing security measures to protect sensitive health information. These advancements promise to enhance clinical operations, facilitating personalized treatment, improving patient outcomes, and optimizing resource allocation. The integration of fog computing in healthcare stands poised to transform care delivery, enhancing efficiency, responsiveness, and patient-centricity. The continued advancement of this technology will be critical in fulfilling the increasing needs of modern healthcare, eventually leading to a more connected and robust healthcare environment.

## References

[1] Elhadad A, Alanazi F, Taloba AI, Abozeid A (2022). Fog computing service in the healthcare monitoring system for managing the real-time notification. J Healthc Eng.

[2] Ahmadi Z, Haghi Kashani M, Nikravan M, Mahdipour E (2021). Fog-based healthcare systems: a systematic review. Multimed Tools Appl.

[3] Shukla S, Hassan MF, Khan MK, Jung LT, Awang A (2019). An analytical model to minimize the latency in healthcare internet-of-things in fog computing environment. PLoS One.

[4] Mudawi NA (2022). Integration of IoT and fog computing in healthcare based the smart intensive units. IEEE Access.

[5] Ortiz-Garcés I, Andrade RO, Sanchez-Viteri S, Villegas-Ch W (2023). Prototype of an emergency response system using IoT in a fog computing environment. Computers.

[6] Tselios C, Politis I, Amaxilatis D, Akrivopoulos O, Chatzigiannakis I, Panagiotakis S, Markakis EK (2022). Melding fog computing and IoT for deploying secure, response-capable healthcare services in 5G and beyond. Sensors (Basel).

[7] Iorga M, Feldman L, Barton R, Martin MJ, Goren NS, Mahmoudi C (2018). Fog Computing Conceptual Model: Recommendations of the National Institute of Standards and Technology. NIST.

[8] Kashyap V, Kumar A, Kumar A, Hu YC (2022). A systematic survey on fog and IoT driven healthcare: open challenges and research issues. Electronics.

[9] Alzoubi YI, Gill A, Mishra A (2022). A systematic review of the purposes of blockchain and fog computing integration: classification and open issues. J Cloud Comput (Heidelb).

[10] Alshuaibi EA, Hamdi AM, Hussain FK (2024). Volunteer computing for fog scalability: a systematic literature review. Internet Things.

[11] Lim J (2021). Scalable fog computing orchestration for reliable cloud task scheduling. Appl Sci.

[12] Alwakeel AM (2021). An overview of fog computing and edge computing security and privacy issues. Sensors (Basel).

[13] Rezapour R, Asghari P, Javadi HHS, Ghanbari S (2021). Security in fog computing: a systematic review on issues, challenges and solutions. Comput Sci Rev.

[14] Quy VK, Hau NV, Anh DV, Ngoc LA (2022). Smart healthcare IoT applications based on fog computing: architecture, applications and challenges. Complex Intell Systems.

[15] Vilela PH, Rodrigues JJ, Righi RD, Kozlov S, Rodrigues VF (2020). Looking at fog computing for e-health through the lens of deployment challenges and applications. Sensors (Basel).

[16] Mani N, Singh A, Nimmagadda SL (2024). An IoT guided healthcare monitoring system for managing real-time notifications by fog computing services. Procedia Comput Sci.

[17] Huang C, Wang J, Wang S, Zhang Y (2023). Internet of medical things: a systematic review. Neurocomputing.

[18] Bhatia J, Italiya K, Jadeja K (2022). An overview of fog data analytics for IoT applications. Sensors (Basel).

[19] Shwe T, Aritsugi M (2024). Optimizing data processing: a comparative study of big data platforms in edge, fog, and cloud layers. Appl Sci.

[20] Kumar A, Gaur N, Nanthaamornphong A (2024). Improving the latency for 5G/B5G based smart healthcare connectivity in rural area. Sci Rep.

[21] Kumar D, Maurya AK, Baranwal G (2021). IoT services in healthcare industry with fog/edge and cloud computing. IoT-Based Data Analytics for the Healthcare Industry.

[22] Hassan SR, Ahmad I, Ahmad S, Alfaify A, Shafiq M (2020). Remote pain monitoring using fog computing for e-healthcare: an efficient architecture. Sensors (Basel).

[23] Rincon JA, Guerra-Ojeda S, Carrascosa C, Julian V (2020). An IoT and fog computing-based monitoring system for cardiovascular patients with automatic ECG classification using deep neural networks. Sensors (Basel).

[24] Ben Hassen H, Ayari N, Hamdi B (2020). A home hospitalization system based on the internet of things, fog computing and cloud computing. Inform Med Unlocked.

[25] Yousefpour A, Fung C, Nguyen T (2019). All one needs to know about fog computing and related edge computing paradigms: a complete survey. J Syst Archit.

[26] Li M, Jiang Y, Zhang Y, Zhu H (2023). Medical image analysis using deep learning algorithms. Front Public Health.

[27] Kraemer FA, Braten AE, Tamkittikhun N, Palma D (2017). Fog computing in healthcare-a review and discussion. IEEE Access.

[28] Nancy AA, Ravindran D, Vincent DR, Srinivasan K, Chang CY (2023). Fog-based smart cardiovascular disease prediction system powered by modified gated recurrent unit. Diagnostics (Basel).

[29] Singh P, Kaur R (2020). An integrated fog and artificial intelligence smart health framework to predict and prevent COVID-19. Glob Transit.

[30] Guo Y, Ganti S, Wu Y (2024). Enhancing energy efficiency in telehealth internet of things systems through fog and cloud computing integration: simulation study. JMIR Biomed Eng.

[31] Kaur I, Saini KS, Khaira JS (2020). Fog integrated novel architecture for telehealth services with swift medical delivery. Fog, Edge, and Pervasive Computing in Intelligent IoT Driven Applications.

[32] Kelkile ME (2023). Optimizing telemedicine framework using fog computing for smart healthcare systems. J Sen Net Data Comm.

[33] Fernández-Caramés TM, Froiz-Míguez I, Blanco-Novoa O, Fraga-Lamas P (2019). Enabling the internet of mobile crowdsourcing health things: a mobile fog computing, blockchain and IoT based continuous glucose monitoring system for diabetes mellitus research and care. Sensors (Basel).

[34] Klonoff DC (2017). Fog computing and edge computing architectures for processing data from diabetes devices connected to the medical internet of things. J Diabetes Sci Technol.

[35] Dadkhah M, Mehraeen M, Rahimnia F, Kimiafar K (2021). Use of internet of things for chronic disease management: an overview. J Med Signals Sens.

[36] Maurya JP, Kumar M, Kumar V (2024). Fog computing and blockchain-based IoMT for personalized healthcare. Emerging Technologies and Security in Cloud Computing.

[37] Jagadeeswari V, Subramaniyaswamy V, Logesh R, Vijayakumar V (2018). A study on medical internet of things and big data in personalized healthcare system. Health Inf Sci Syst.

[38] Jeyashree G, Padmavathi S (2023). A fog cluster-based framework for personalized healthcare monitoring. Research Advances in Network Technologies.

[39] Jain S, Naicker D, Raj R, Patel V, Hu YC, Srinivasan K, Jen CP (2023). Computational intelligence in cancer diagnostics: a contemporary review of smart phone apps, current problems, and future research potentials. Diagnostics (Basel).

[40] Welhenge A (2022). Deep learning based breast cancer detection system using fog computing. J Discrete Math Sci Cryptogr.

[41] Pati A, Parhi M, Pattanayak BK, Sahu B, Khasim S (2023). CanDiag: fog empowered transfer deep learning based approach for cancer diagnosis. Designs.

[42] Li F, Shankar A, Santhosh Kumar B (2021). Fog-internet of things-assisted multi-sensor intelligent monitoring model to analyze the physical health condition. Technol Health Care.

[43] Cheikhrouhou O, Mershad K, Jamil F, Mahmud R, Koubaa A, Moosavi SR (2023). A lightweight blockchain and fog-enabled secure remote patient monitoring system. Internet Things.

[44] Jayson Baucas M, Spachos P (2020). Fog and IoT-based remote patient monitoring architecture using speech recognition. 2020 IEEE Symposium on Computers and Communications (ISCC).

[45] Akrivopoulos O, Chatzigiannakis I, Tselios C, Antoniou A (2017). On the deployment of healthcare applications over fog computing infrastructure. 2017 IEEE 41st Annual Computer Software and Applications Conference (COMPSAC).

[46] Medina J, Espinilla M, Zafra D, Martínez L, Nugent C (2017). Fuzzy fog computing: a linguistic approach for knowledge inference in wearable devices. Ubiquitous Computing and Ambient Intelligence.

[47] Borthakur D, Dubey H, Constant N, Mahler L, Mankodiya K (2017). Smart fog: fog computing framework for unsupervised clustering analytics in wearable internet of things. 2017 IEEE Global Conference on Signal and Information Processing (GlobalSIP).

[48] Akrivopoulos O, Amaxilatis D, Mavrommati I, Chatzigiannakis I (2019). Utilising fog computing for developing a person-centric heart monitoring system. J Ambient Intell Smart Environ.

[49] Paul A, Pinjari H, Hong WH, Seo HC, Rho S (2018). Fog computing-based IoT for health monitoring system. J Sensors.

[50] Anand D, Khemchandani V (2020). Data security and privacy functions in fog computing for healthcare 4.0. Fog Data Analytics for IoT Applications: Next Generation Process Model With State of the Art Technologies.

[51] Thota C, Sundarasekar R, Manogaran G, Varatharajan R, Priyan MK (2018). Centralized fog computing security platform for IoT and cloud in healthcare system. Fog Computing: Breakthroughs in Research and Practice.

[52] Mehdipour F, Javadi B, Mahanti A, Ramirez-Prado G (2019). Fog computing realization for big data analytics. Fog and Edge Computing: Principles and Paradigms.

[53] Moysiadis V, Sarigiannidis P, Moscholios I, Honeine P (2018). Towards distributed data management in fog computing. Wirel Commun Mob Comput.

[54] Alekseeva D, Ometov A, Arponen O, Lohan ES (2022). The future of computing paradigms for medical and emergency applications. Comput Sci Rev.

[55] Alnaim AK, Alwakeel AM (2023). Machine-learning-based IoT-edge computing healthcare solutions. Electronics.

[56] Dash Y, Jajoria P (2023). Fog computing: applications in smart healthcare. 2023 3rd International Conference on Advancement in Electronics & Communication Engineering (AECE).

[57] Losavio M (2020). Fog computing, edge computing and a return to privacy and personal autonomy. Procedia Comput Sci.

[58] Pinto GP, Prazeres C (2024). Towards data privacy in a fog of things. Internet Technol Lett.

[59] Yang R, Xu Q, Au MH, Yu Z, Wang H, Zhou L (2018). Position based cryptography with location privacy: a step for fog computing. Future Gener Comput Syst.

[60] Ni J, Zhang K, Lin X, Shen X (2018). Securing fog computing for internet of things applications: challenges and solutions. IEEE Commun Surv Tutor.

[61] Sadri AA, Rahmani AM, Saberikamarposhti M, Hosseinzadeh M (2021). Fog data management: a vision, challenges, and future directions. J Netw Comput Appl.

[62] Mukherjee M, Ferrag MA, Maglaras L, Derhab A, Aazam M (2020). Security and privacy issues and solutions for fog. Fog and Fogonomics.

[63] Khalid T, Abbasi MAK, Zuraiz M (2021). A survey on privacy and access control schemes in fog computing. Int J Commun Syst.

[64] Liu JN, Weng J, Yang A, Chen Y, Lin X (2020). Enabling efficient and privacy-preserving aggregation communication and function query for fog computing-based smart grid. IEEE Trans Smart Grid.

[65] Das R, Inuwa MM (2023). A review on fog computing: issues, characteristics, challenges, and potential applications. Telemat Inform Rep.

